# Comparison of the quality of dried persimmon (*Diospyros kaki* THUNB.) treated with medicinal plant extracts and food additives

**DOI:** 10.1002/fsn3.673

**Published:** 2018-10-20

**Authors:** Jun‐Hoi Kim, Il Kyung Chung, Hak Yoon Kim, Kyung‐Min Kim

**Affiliations:** ^1^ School of Applied Biosciences College of Agriculture & Life Science Kyungpook National University Daegu Korea; ^2^ Department of Biotechnology Catholic University of Daegu Gyeongsan‐Si Gyeongbuk Korea; ^3^ Department of Global Environment Keimyung University Daegu Korea

**Keywords:** drying, extraction/separation, food quality, food safety

## Abstract

We evaluated the direct application of different extracts from plant‐derived compounds at different ratios. The best effect was observed with the combination of 18.18% clove, 9.90% cinnamon, 9.09% licorice, 4.55% firmament, 4.55% grapefruit seed extract, and 54.54% apple cider vinegar. The combination of these compounds improved the moisture content of the fruit and showed antifungal, antibrowning, and antifungal/antibrowning effects as compared with the control following 6‐week treatment. The treatment resulted in an increase in the overall sugar concentration of dried persimmons. Antibrowning/antifungal test showed high sugar content of 30–39 °brix. The hardness of the treatment groups was similar to that of the control and decreased by 0.5 to 0.8 after 6 weeks. The evaluation of color change revealed a decreasing tendency in the value of △*E* during the drying period. Thus, natural extracts effectively suppressed the quality degradation during drying of persimmon and may be used to replace sulfur fumigation.

## 1 INTRODUCTION

The fruit persimmon (*Diospyros kaki* THUNB.) has been domestically grown for a long time everywhere except mountainous area and regions with an average annual temperature of less than 10°C. Persimmon is produced mainly in temperate Asia region such as South Korea, China, and Japan (Song, Choo, Kim, & Kang, 2005). The main component of this fruit is a saccharide comprising glucose and fructose; the saccharide content slightly differs between sweet and astringent persimmon. In addition, persimmon is a rich source of minerals as well as vitamins A, B, and C and also contains pectin and carotenoid. The color of the fruit is attributed to the carotenoid pigments in the pericarp and the content of lycopene (dark orange) is related with the autumn daylight conditions. Persimmon contains variety of active compounds such as carotenoid, tannin, flavonoid, terpenoid, steroid, and naphthoquinone and is rich in sugars, amino acids, minerals, and lipids (Kang et al., 2004).

According to Donguibogam and Botanical list, persimmon is sweet, nontoxic, and frequently used to cure diseases such as boils, inflammation, furuncles, and burn. In addition, the fruit is known to assist in digestion and viscera activation and displays strong protective effects against diarrhea, diuresis, hemostasis, hangover, cough, bronchitis, and hypertension (Kang et al., 2004; Kim, 2005; Kim, Kang, & Kim, 2005; Moon et al., 2004; Park et al., 2006). Persimmon is classified as sweet and astringent persimmon. Diospyrin, a type of tannin, imparts astringency to persimmon and its water‐soluble nature may impart an evident bitter taste. In combination with acetaldehyde, it changes into its insoluble form that suppresses the bitter taste (Kang et al., 2004). Sweet persimmon is usually eaten raw, but astringent persimmon is used to make ripen persimmon, dried persimmon, and other products. Dried persimmon displays the advantage of long‐term storage; more than 50% astringent persimmon is processed into dried persimmon (Hong et al., 2001; Park et al., 2006). Studies on astringent persimmon have described the qualitative differences in persimmon tannin and natural methods for the removal of astringency as well as the physiological/functional characteristics of tannins isolated from astringent persimmon (Seo, Jeong, & Kim, 2000; Seong & Han, 1999). Drying persimmon increases the sweetness and vitamin A content and is an important method for extending the availability period of persimmon. Dried persimmon has rich flavor and specific physical properties. Dried persimmon may be classified into dried and semidried persimmon based on its water content. In general, dried persimmon is more distributed than its semidried form because semidried persimmon containing high moisture is easily contaminated by fungus, reducing its storage and distribution period. However, there is an increase in the consumption of semidried persimmon in recent years (Hong et al., 2001). The quality of dried persimmon is dramatically affected by the drying method (Moon, Kim, & Sohn, 1993), quality of persimmon, storage and packing method, and local quality characteristics (Kim, Kang, et al., 2004). Dried persimmon is difficult to store at room temperature, owing to its susceptibility to fungal contamination. Dried persimmon is usually produced by solar drying method, which may cause deterioration through the incorporation of foreign matter, discoloration, and fungal contamination. During storage and distribution, the quality of dried persimmon may be rapidly decreased owing to several factors such as fungal contamination, structural hardening, sugar discoloration, powdering, and browning (Kim, Kang, et al., 2004). Moon, Lee, Kim, and Kim (1996) observed dried persimmon produced domestically and confirmed the presence of white powder on its surface with an electron microscope. The white powder was found to be crystallized glucose from the sugar present inside the flesh. This sugar moved to the surface with moisture and dried to form powder during the drying period. The contamination by microorganisms during drying, storage, and distribution may cause fruit deterioration and is known to be unhygienic. To prevent deterioration during the drying period for improved storage, many farmers use sulfurate after peeling. However, there are safety concerns related to the harmful effects of sulfur dioxide produced from the combustion of sulfur (Im & Lee, 2007). The use of active components separated from medicinal plants is rapidly increasing in recent years to prevent and cure various human diseases. The study of natural bioactive substances that are secondary metabolites is gaining interest. As bioactive substances display strong activities at relatively small amounts, regulation is reinforced on their use, owing to safety problems caused by some artificial synthetic products. The increase in human safety and health issues has highlighted the need for the extraction of bioactive substances from natural sources to replace artificial synthetic products (Heo et al., 2011; Kim, Hong, & Shin, 2012; Kim, Kwon, Cho, & Lee, 2012).

At present, several studies are focusing on the identification of compounds with high antioxidative activities and several health benefits from natural sources such as plants in the form of medicines and health supplements (Lee, Oh, & Hong, 2002; Shin, Kim, & Han, 1997). Molecules such as α‐tocopherol, vitamin C, carotenoid, and flavonoid are known for their natural antioxidant activities and are usually extracted from animals and plants. Secondary metabolites from plants that prevent cell damage through the removal or suppression of active oxide are identified (Jung et al., 2004). According to recent reports, most natural antioxidants are derived from plants and usually identified as polyphenol components (Pratt, 1992). Flavonoids are known to prevent lipid oxidation, remove active oxides, and reduce oxidative stress. Flavonoids are widely used in food, medicines, and cosmetics to prevent or delay cancer, antiaging, heart diseases, etc. (Kim et al., 2005). Studies on antibiotic substances from natural sources have been performed for a long time (Ha, Lee, & Kang, 2000; Kim, Shu, & Chung, 2006) and include investigation of natural preservatives and antibiotics from herbs and spices. In addition, antibiotics from natural sources are used to suppress fungal parasites from food and human body (Lee et al., 2002; Shin et al., 1997). Garlic, onion, *Thymus vulgaris* (Buchanan & Shepherd, 1981; Montes‐Belmont & Carvajal, 1998; Yin & Cheng, 1998), clove, cinnamon, *Paeonia suffruticosa* (Hwang & Han, 2003), *Salvia miltiorrhiza* Bunge (Choi & Han, 2003), *Rhodiola sachalinensis* (Choi & Han, 2003), and *Morus alba* root barks (Shim et al., 2004) have been used for the extraction of natural antibiotics and to extend storage life of several products through their antibiotic effects. Studies have reported the effects of pretreatment with grapefruit seed extract and cinnamon on the quality of dried persimmon (Park, Kim, Kim, Kim, & Chang, 1995; Park, Lee, Cha, Kim, & Kim, 2005; Park et al., 2006). Hong et al. (2001) reported changes in the microflora of dried persimmon during processing, preservation, manufacturing, and storage. The longer storage time was yeast and acetic acid reduced. Microbial contamination during drying of persimmon was detected using a variety of media. *Penicillium* was the most widely detected fungus. During the manufacturing and storage of persimmon, contamination with *Penicillium* and bacteria has been reported (Kim, Park, Lee, Kim, & Cha, 2004; Kwon, Jeong, Hong, Chae, & Park, 2006). In recent years, studies have reported the identification and prevention of microbial contamination. However, there are several shortcomings in the established methods to prevent contamination. In this direction, we investigated the effected of natural extracts and food additives on the quality of dried persimmons.

## 2 MATERIALS AND METHODS

### 2.1 Persimmon materials and treatment

Persimmons produced in 2012 were used for the antibrowning, antifungal, and antibrowning/antifungal experiments. The samples were subjected to the extraction method as described below. To obtain 5% plant extract, 50 g sample was extracted in 950 ml hot water for 2 hr, followed by centrifugation at 120 x g for 20 min. The plant‐derived compounds with effective antibrowning activity used were as follows: citric acid, vitamin C, citric acid + vitamin C, potassium pyrosulfate, sodium chloride, cysteine, acetic acid, grapefruit seed extract, and natural products (*Glycyrrhiza uralensis*,* Cinnamomum cassia*,* Syzygium aromaticum*, and *Cnidium officinale*). The concentration of compounds was as follows: citric acid 3%, vitamin C 3%, potassium pyrosulfate 3%, sodium chloride 3%, cysteine 0.3%, acetic acid 1%, and extract of grapefruit seed 1%. The extraction method for four natural products was the same as mentioned above. After peeling, persimmon was soaked in 50 ml extraction liquid and air‐dried, followed by the investigation of its food quality characteristics.

### 2.2 Unique number and ingredient mark for antibrowning

Medicinal plant extract and food additive mixing rate for dried persimmon browning prevention effect experiment. The numbers and components are as follows: 1; Control(untreated control plot), 2; True Control (distilled water), 3; 3% Citric acid, 4; 1% Vitamin C, 5; 3% Citric acid +1% Vitamin C, 6; 1% Potassium meta‐bisulfite, 7; 1% acetic acid, 8; 3% acetic acid, 9; 1% grapefruit seed extract, 10; 5% chrysanthemum indicum, 11; 5% licorice, 12; 5% green tea, 13; 5% cinnamon, 14; 5% clove, 15; 5% cnidium, 16; 5% turmeric, 17; 2% polyphosphate, 18; Cider vinegar (Acidity of about 5%), 19; mix proportion A = Cider vinegar 50: 3% Citric acid 20: 5% licorice 20: 5% cinnamon 10, 20; mix proportion B = Cider vinegar 60: 3% Citric acid 10: 5% licorice 20: 5% cinnamon 10, and 21; mix proportion C = Cider vinegar 70: 3% Citric acid 10: 5% licorice 10: 5% cinnamon 10.

### 2.3 Unique number and ingredient mark for antifungal

Medicinal plant extract and food additive mixing rate for dried persimmon antibacterial effect experiment. The numbers and components are as follows: 1; Control(untreated control plot), 2; True Control (distilled water), 22; 3% Citric acid +1% Vitamin C, 23; 1% Potassium meta‐bisulfite, 24; 1% acetic acid, 25; 3% acetic acid, 26; 5% acetic acid, 27; 3% grapefruit seed extract, 28; 5% grapefruit seed extract, 29; 2% polyphosphate, 30; 50% ethanol cinnamon extract, 31; 50% ethanol licorice extract, 32; 50% ethanol clove extract, 33; 50% ethanol cnidium extract, 34; Cider vinegar (Acidity of about 5%), 35; mix proportion A = Cider vinegar 50: cinnamon extract 20: 5% grapefruit seed extract 10: licorice 10: cnidium 5.5: clove 4.5, 36; mix proportion B = Cider vinegar 50: clove extract 20: 5% grapefruit seed extract 10: cinnamon 10: licorice 5.5: cinnamon 4.5, and 37; mix proportion C = Cider vinegar 70: cinnamon extract 15: licorice 10: cnidium 4.5: clove 0.5.

### 2.4 Unique number and ingredient mark for antibrowning/antifungal

Medicinal plant extract and food additive mixing rate for dried persimmon browning prevention effect/antifungal effect experiment. The numbers and components are as follows: 38; mix proportion final A = Cider vinegar 50: 3% Citric acid 20: 5% grapefruit seed extract 10: cinnamon 10: licorice 5: cnidium 4.9: clove 0.1, and 39; mix proportion final B = Cider vinegar 60: 3% Citric acid 15: 5% grapefruit seed extract 10: cinnamon 10: licorice 2.5: cnidium 2.45: clove 0.05.

### 2.5 Persimmon quality investigation and statistics

In total, 2,340 persimmons were collected from each area. The number used in the treatment was 39, of which there were nine combinations. These were hung on the drying racks similar to those used in Korean farms. Ten dried persimmons were randomly selected from each region. The investigation was conducted six times every week. Briefly, 5.0–5.5 g persimmon samples were evaluated at 110°C for 60 min. The moisture content was analyzed with a moisture analyzer (MB45 Moisture Analyzer, Ohaus, U.S.A.). An electronic balance (Adventurer Pro, AVG264C, Ohaus, U.S.A.) was used for the investigation of persimmon weight. The values of several parameters were read to the second decimal place. A refraction saccharometer (Master‐10T, Atago, No.2372, Japan and Master‐20T, Atago, No.2382, Japan) was used for the investigation of the sugar content. Persimmon samples were homogenized in distilled water at a ratio of 1:3. A hardness testing machine (FHM‐5, Takemura, Japan) was used to evaluate the hardness of dried persimmons samples. A color difference meter (J801S, Color Techno System, Japan) was used to measure the chromaticity of dried persimmon. Dried persimmons were cut in two halves and spread on petri dishes. SPSS (ver. 20) was used to calculate the average as well as standard deviation from each experimental result and *t*‐test and one‐way analysis of variance (ANOVA) were used to analyze the significance of mean.

## 3 RESULTS AND DISCUSSION

**Figure 1:**
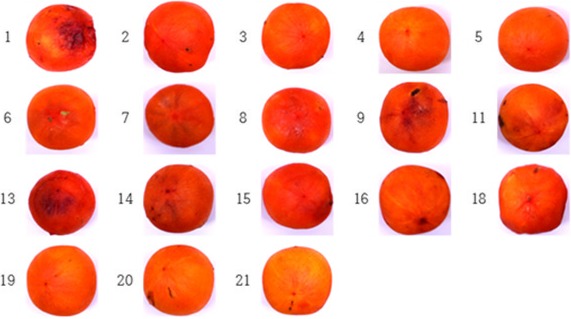
Antibrowning effects of medicinal plant extracts and food additives on dried persimmons. 1: Control(untreated control plot), 2: True Control (distilled water), 3: 3% Citric acid, 4: 1% Vitamin C, 5: 3% Citric acid +1% Vitamin C, 6: 1% Potassium meta‐bisulfite, 7: 1% acetic acid, 8: 3% acetic acid, 9: 1% grapefruit seed extract, 10: 5% chrysanthemum indicum, 11: 5% licorice, 12: 5% green tea, 13: 5% cinnamon, 14: 5% clove, 15: 5% cnidium, 16: 5% turmeric, 17: 2% polyphosphate, 18: Cider vinegar (Acidity of about 5%), 19: mix proportion A = Cider vinegar 50: 3% Citric acid 20: 5% licorice 20: 5% cinnamon 10, 20: mix proportion B = Cider vinegar 60: 3% Citric acid 10: 5% licorice 20: 5% cinnamon 10, 21: mix proportion C = Cider vinegar 70: 3% Citric acid 10: 5% licorice 10: 5% cinnamon 10

The treatment with different combinations of medicinal plant extracts and food additives showed different degree of antibrowning activity (Table 1). As shown in Figure 1, combinations 19, 20, and 21 were confirmed to display good color.

**Table 1:** Changes in the water content with antibrowning, antifungal, and antibrowning/antifungal treatments during 6‐week drying of persimmon. C: control, numbers are according to materials and method

Solution	Water content (%)					
	1 week	2 weeks	3 weeks	4 weeks	5 weeks	6 weeks
1	60.5 ± 2.8	40.7 ± 17.9	65.1 ± 3.1	68.7 ± 8.7	75.4 ± 3.8	71.0 ± 2.3b
19	60.5 ± 1.9	58.3 ± 15.3	67.8 ± 6.7	65.0 ± 3.6	77.5 ± 4.2	73.2 ± 2.9a
20	60.9 ± 4.0	62.5 ± 5.9	66.1 ± 2.2	69.8 ± 6.6	78.9 ± 6.4	71.8 ± 2.1b
21	59.4 ± 3.5	63.1 ± 1.0	67.9 ± 3.6	68.2 ± 1.8	79.2 ± 2.6	75.8 ± 1.5a
35	61.0 ± 3.3	66.9 ± 3.4	61.6 ± 4.7	66.8 ± 1.7	75.7 ± 2.9	72.8 ± 1.8b
36	62.7 ± 2.0	65.2 ± 1.6	63.7 ± 3.6	67.2 ± 0.7	78.7 ± 1.3	75.5 ± 3.5a
37	61.1 ± 3.7	67.2 ± 3.2	65.9 ± 2.0	65.0 ± 2.6	76.3 ± 6.5	73.4 ± 2.2a
38	59.6 ± 1.7	68.1 ± 3.3	63.5 ± 3.2	66.2 ± 0.7	75.1 ± 4.8	76.1 ± 2.9a
39	61.2 ± 3.8	72.3 ± 6.2	64.2 ± 4.8	66.4 ± 3.1	77.0 ± 1.0	73.2 ± 2.0a

Results are presented as means ± standard deviation of three replicates. DMRT analysis was performed at 6 weeks.

**Figure 2:**
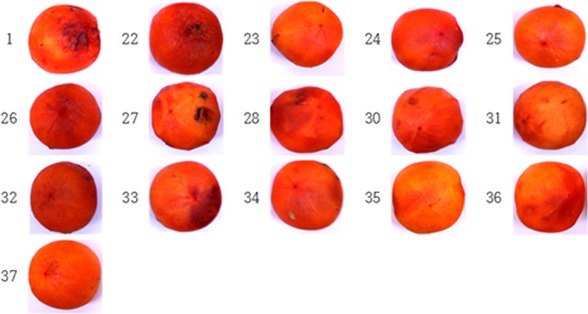
Antifungal effects of medicinal plant extracts and food additives on dried persimmons. 1: Control(untreated control plot), 22: 3% Citric acid +1% Vitamin C, 23: 1% Potassium meta‐bisulfite, 24: 1% acetic acid, 25: 3% acetic acid, 26: 5% acetic acid, 27: 3% grapefruit seed extract, 28: 5% grapefruit seed extract, 29: 2% polyphosphate, 30: 50% ethanol cinnamon extract, 31: 50% ethanol licorice extract, 32: 50% ethanol clove extract, 33: 50% ethanol cnidium extract, 34: Cider vinegar (Acidity of about 5%), 35: mix proportion A = Cider vinegar 50: cinnamon extract 20: 5% grapefruit seed extract 10: licorice 10: cnidium 5.5: clove 4.5, 36: mix proportion B = Cider vinegar 50: clove extract 20: 5% grapefruit seed extract 10: cinnamon 10: licorice 5.5: cinnamon 4.5, 37: mix proportion C = Cider vinegar 70: cinnamon extract 15: licorice 10: cnidium 4.5: clove 0.5

The treatment with different combinations of medicinal plant extracts and food additives resulted in different degree of antifungal activity (Table 2). As shown in Figure 2, combinations 35, 36, and 37 were confirmed to show higher antifungal activity than other combinations.

**Table 2:** Changes in the sugar content with antibrowning, antifungal, and antibrowning/antifungal treatments during 6‐week drying of persimmon. C: control, numbers are according to materials and method

Solution	Sugar content (^◦^brix)					
	1 week	2 weeks	3 weeks	4 weeks	5 weeks	6 weeks
1	20.9 ± 0.3	23.8 ± 0.8	27.2 ± 2.5	27.7 ± 4.9	27.7 ± 0.5	35.8 ± 4.7a
19	19.0 ± 2.4	22.1 ± 2.4	25.2 ± 4.2	29.6 ± 3.2	31.7 ± 4.1	34.2 ± 2.5b
20	20.6 ± 2.0	20.1 ± 2.9	25.4 ± 3.2	28.0 ± 1.7	31.1 ± 2.0	37.6 ± 1.9a
21	18.1 ± 0.2	20.0 ± 1.3	23.9 ± 3.8	25.9 ± 5.9	28.5 ± 3.7	33.1 ± 1.7b
35	20.3 ± 1.2	20.4 ± 0.6	24.5 ± 2.7	32.3 ± 1.5	29.9 ± 2.4	28.8 ± 2.5c
36	20.9 ± 1.4	22.0 ± 1.3	38.5 ± 15.4	28.3 ± 6.6	28.2 ± 0.8	32.1 ± 3.0b
37	16.0 ± 1.0	20.1 ± 2.0	24.8 ± 2.6	31.4 ± 0.6	33.3 ± 5.3	32.2 ± 2.0b
38	18.8 ± 2.1	21.5 ± 1.1	25.0 ± 3.8	28.7 ± 3.3	30.1 ± 2.3	34.6 ± 2.5a
39	17.6 ± 1.4	24.3 ± 1.8	25.2 ± 4.4	29.5 ± 3.2	29.8 ± 3.5	31.3 ± 1.1c

Results are presented as means ± standard deviation of three replicates. DMRT analysis was performed at 6 weeks.

The treatment with different combinations of medicinal plant extracts and food additives showed different degrees of antibrowning and antifungal activity (data not shown). Combinations 38 and 39 were confirmed to show higher antibrowning and antifungal activity as compared with other combinations and △*E* representing chromaticity was nontreated, at 38, 60.8 and at 39, 50.6.

The moisture content of dried persimmon was evaluated following treatment with different combinations of medicinal plant extracts and food additives for 6 weeks (Table 1). The water content was the highest for the treatment group (75.8%). After 6 weeks, the water content increased as compared to that observed after 1 week, while the control showed relatively lower moisture content as compared with the treatment groups. The water content of the control group was higher than that of the treatment groups during early days but decreased in a time‐dependent manner. Kim, Kang, et al. (2004) reported the water content distribution of dried persimmon of Sangju to be 32.23%–38.20%. Here, we observed around 70% moisture content in semidried persimmon after 6 weeks. This is judged that very suitable semidried persimmon quality because it is similar to the typical moisture content of 45%–50%.

We investigated the sugar content of the dried persimmons after treatment with medicinal plant extracts and food additives for 6 weeks (Table 2). The samples subjected to treatment showed an increase in the overall sugar content with an increase in the drying period. The sugar content of untreated persimmon was about 20 °brix, which increased to about 30–37 °brix after drying. The highest sugar content was reported to be 38 °brix in treatment condition 20. Kim, Kang, et al. (2004) reported a sugar content of 55 °brix for dried persimmon of Sangju area. These variations in the results may be associated with the differences in the production environment as well as the drying conditions employed.

The overall hardness of persimmon was lower throughout the drying period following treatment with medicinal plant extracts and food additives (Table 3). At 6 weeks, the hardness was higher in treatment 20, 6 weeks after on average treatment 37's hardness was lessen about 0.5, so hardness go higher than the first as a whole. According to Lee et al. (1994), between drying period 5 days and 20 days, hardness was so soft below 0.5 kg/mm^2^ that the result was similar.

**Table 3:** Changes in hardness with antibrowning, antifungal, and antibrowning/antifungal treatments and during 6‐week persimmon drying. C: control, numbers are according to materials and method. It is the period that represents the best value of the product in dried persimmon

Solution	Hardness (kg/mm^2^)					
	1 week	2 weeks	3 weeks	4 weeks	5 weeks	6 weeks
1	2.2 ± 2.3	1.1 ± 0.3	0.9 ± 0.2	0.9 ± 0.1	0.6 ± 0.1	0.8 ± 0.1a
19	1.2 ± 0.3	1.2 ± 0.1	0.9 ± 0.2	0.9 ± 0.1	0.5 ± 0.1	0.7 ± 0.1b
20	1.2 ± 0.4	1.2 ± 0.4	0.8 ± 0.1	1.0 ± 0.2	0.2 ± 0.0	0.8 ± 0.1a
21	1.4 ± 0.2	1.5 ± 0.2	0.9 ± 0.1	1.0 ± 0.2	0.5 ± 0.1	0.8 ± 0.1a
35	1.1 ± 0.2	0.7 ± 0.1	0.8 ± 0.2	1.2 ± 0.2	0.4 ± 0.1	0.7 ± 0.1b
36	0.9 ± 0.2	1.0 ± 0.2	0.8 ± 0.0	1.3 ± 0.6	0.5 ± 0.1	0.7 ± 0.0b
37	1.1 ± 0.1	1.0 ± 0.3	0.8 ± 0.1	0.8 ± 0.1	0.5 ± 0.2	0.6 ± 0.0b
38	1.1 ± 0.2	0.9 ± 0.1	0.8 ± 0.1	1.3 ± 0.5	1.0 ± 0.0	0.7 ± 0.3b
39	1.2 ± 0.2	0.8 ± 0.4	0.9 ± 0.2	0.9 ± 0.3	0.3 ± 0.0	0.8 ± 0.2a

Results were presented as means ± standard deviation of three replicates. DMRT analysis was performed at 6 weeks.

Following treatment with medicinal plant extracts and food additives (Table 4), the color change of dried persimmon was monitored weekly. Treatment conditions 19, 20, and 21 resulted in the change in color value and showed a decreasing tendency during the drying period. The values of *L* and *a* increased between 1 week and 3 weeks and decreased thereafter. The value of *L* indicates brightness; lightest color was observed in 3 weeks and the color darkened thereafter. The value of *a* is indicative of the red color and was high until 3 weeks and further increased after 3 weeks. Values of *b* and △*E* reduced following treatment. The value of *b* is indicative of the yellow color; thus, the yellow color of dried persimmon decreased with time. △*E* represents color difference with time. Treatment conditions 35, 36, and 37 induced a change in △*E* value, showing a tendency to decrease during the drying period. *L* value increased until 3 weeks and decreased thereafter. *L* value was the lowest during 3 weeks and increased thereafter.

**Table 4:** Changes in the color difference with antibrowning, antifungal, and antibrowning/antifungal treatments during 4‐6 week drying of persimmon. C: control, numbers are according to materials and method

Solution	Color difference											
	1 week				2 weeks				3 weeks			
	*L*	*a*	*b*	∆*E*	*L*	*a*	*b*	∆*E*	*L*	*a*	*b*	∆*E*
1	1.4 ± 13.9	6.3 ± 3.7	70.0 ± 17.7	71.1 ± 18.7	36.6 ± 7.1	14.0 ± 6.1	51.0 ± 16.8	69.6 ± 21.1	51.1 ± 3.5	17.8 ± 1.7	61.2 ± 5.9	81.7 ± 6.2
19	4.9 ± 2.4	3.9 ± 1.8	73.6 ± 3.1	73.9 ± 3.2	44.5 ± 6.8	10.9 ± 2.2	47.5 ± 9.9	66.0 ± 12.1	51.2 ± 9.3	16.1 ± 1.5	59.8 ± 9.5	80.4 ± 12.6
20	19.3 ± 2.1	8.3 ± 1.6	91.7 ± 1.8	94.1 ± 2.1	44.6 ± 6.0	8.2 ± 3.6	47.8 ± 10.0	65.9 ± 11.8	46.6 ± 7.4	12.0 ± 3.6	51.1 ± 12.8	70.2 ± 14.8
21	9.6 ± 3.0	10.1 ± 1.8	82.2 ± 2.3	83.4 ± 2.7	39.6 ± 30.8	9.1 ± 7.1	56.8 ± 16.8	77.7 ± 19.3	39.9 ± 4.1	9.2 ± 1.9	43.2 ± 3.8	59.7 ± 5.5
35	5.4 ± 9.9	9.8 ± 2.7	76.2 ± 13.9	77.3 ± 15.0	48.2 ± 7.5	9.0 ± 3.3	49.5 ± 10.4	69.7 ± 13.0	57.1 ± 4.5	16.4 ± 1.6	67.5 ± 5.5	89.9 ± 7.2
36	−1.0 ± 4.0	5.0 ± 3.0	66.6 ± 5.5	67.0 ± 5.7	45.0 ± 2.9	9.8 ± 2.1	50.0 ± 6.0	68.0 ± 6.5	39.2 ± 7.4	26.8 ± 37.9	41.0 ± 9.5	57.4 ± 12.3
37	7.8 ± 4.2	7.5 ± 5.3	78.1 ± 9.1	79.0 ± 9.8	46.6 ± 2.2	8.4 ± 0.8	51.0 ± 4.3	69.6 ± 4.7	45.9 ± 5.0	12.7 ± 2.9	51.9 ± 8.3	51.4 ± 28.9
38	6.6 ± 2.6	4.4 ± 1.9	73.1 ± 3.3	73.6 ± 2.9	43.2 ± 8.4	9.9 ± 1.5	46.4 ± 10.3	44.2 ± 21.6	40.3 ± 4.6	6.4 ± 1.7	43.4 ± 7.8	59.6 ± 9.0
39	10.0 ± 7.2	5.5 ± 2.4	79.1 ± 10.0	80.1 ± 10.8	42.4 ± 7.4	4.9 ± 2.8	40.2 ± 8.4	58.6 ± 11.1	45.4 ± 6.7	12.0 ± 5.1	51.5 ± 11.0	69.8 ± 13.3

Solution	Color difference											
	4 weeks				5 weeks				6 weeks			
	*L*	*a*	*b*	∆*E*	*L*	*a*	*b*	∆*E*	*L*	*a*	*b*	∆*E*
1	42.7 ± 8.6	16.4 ± 2.1	53.7 ± 14.4	70.5 ± 16.8	43.0 ± 7.3	9.8 ± 4.7	51.5 ± 11.8	67.9 ± 14.2	30.7 ± 5.2	2.6 ± 2.9	41.2 ± 7.0	51.5 ± 8.8b
19	41.5 ± 4.0	13.5 ± 1.6	54.1 ± 3.8	69.5 ± 5.4	41.5 ± 7.0	12.7 ± 9.1	53.2 ± 14.3	75.7 ± 24.9	25.2 ± 1.2	3.4 ± 1.6	36.5 ± 3.2	48.2 ± 7.8b
20	43.2 ± 3.6	9.4 ± 2.2	51.0 ± 4.2	67.5 ± 5.6	40.1 ± 12.0	3.6 ± 2.1	38.6 ± 4.2	51.7 ± 4.3	26.4 ± 1.2	0.9 ± 3.9	34.2 ± 7.4	43.6 ± 5.0b
21	44.1 ± 2.9	12.0 ± 3.0	55.5 ± 7.5	71.9 ± 8.0	34.1 ± 2.0	2.2 ± 2.2	36.8 ± 4.2	50.3 ± 4.5	27.3 ± 1.6	0.5 ± 1.6	37.3 ± 1.2	16.3 ± 0.8c
35	46.4 ± 3.1	14.4 ± 5.5	59.7 ± 5.2	77.0 ± 6.9	39.8 ± 3.8	6.9 ± 1.9	47.4 ± 5.9	62.2 ± 7.0	32.1 ± 5.9	2.4 ± 5.0	43.4 ± 14.9	54.2 ± 15.6a
36	43.0 ± 2.7	10.3 ± 1.4	55.0 ± 5.6	71.2 ± 6.0	39.4 ± 5.0	9.1 ± 1.4	49.7 ± 7.3	64.1 ± 8.8	27.8 ± 1.8	0.1 ± 1.3	37.6 ± 1.8	46.7 ± 2.5b
37	36.5 ± 7.4	9.4 ± 3.6	41.4 ± 9.8	56.1 ± 12.2	45.3 ± 4.3	8.4 ± 3.2	56.6 ± 6.0	73.0 ± 7.6	23.2 ± 1.3	2.8 ± 2.0	32.1 ± 4.6	39.8 ± 4.5bc
38	45.9 ± 4.8	11.2 ± 1.7	56.1 ± 4.6	73.6 ± 6.3	38.4 ± 7.4	5.3 ± 1.5	51.3 ± 8.7	62.2 ± 9.6	36.2 ± 7.0	2.7 ± 1.7	48.8 ± 8.8	60.8 ± 11.3a
39	48.3 ± 9.0	11.7 ± 3.6	58.6 ± 15.5	70.2 ± 14.5	42.6 ± 3.2	6.1 ± 3.6	53.1 ± 8.5	68.4 ± 8.8	31.5 ± 6.0	2.3 ± 1.0	39.5 ± 8.2	50.6 ± 10.2b

L, Lightness; a, redness; b, yellowness; ∆*E*, ∆E*ab. Results are presented as means ± standard deviation of three replicates. DMRT analysis was performed at 6 weeks.

The value of *a* increased until 4 weeks and then decreased from 4 weeks. The red color observed during first 4 weeks became increasingly darker after 3 weeks. The value of *b* initially decreased but suddenly increased from 4 weeks and decreased thereafter. Upon drying, *b* values gradually disappeared. △*E* values were mostly reduced over time. As observed in treatment conditions 38 and 39, the changes in △*E* decreased during drying. The value of *L* and *a* increased from 1 week to 4 weeks and decreased after 4 weeks. The value of *L* shows the change in the brightness of the treated dried persimmon, indicated by the darker color of the fruit after 4 weeks. The value of *a* showed red color until 4 weeks and changed to dark red after 4 weeks. The values of *b* and △*E* gradually decreased. The value of *b* showed that the yellow color of dried persimmons gradually changed and became fainter with time. The value of △*E* decreased with time and the treated plot showed lower values for the treatment group as compared with control after 3 days of drying. According to Lee et al. (1994), treated dried persimmons showed a gradual decrease in the color difference, similar to the observations of the present study. This may be attributed to the absorption of natural compounds from clove, licorice, or other substances on the surface of dried persimmons.

## 4 CONCLUSIONS

The combination of natural bioactive compounds that showed excellent activity included 18.18% clove, 9.90% cinnamon, 9.09% licorice, 4.55% firmament, 4.55% grapefruit seed extract, and 54.54% apple cider vinegar. The combination of these compounds improved the moisture content of the fruit and showed antifungal, antibrowning, and antifungal/antibrowning effects. Thus, natural extracts effectively suppress the quality degradation during drying of persimmon and may be used to replace sulfur fumigation.

## CONFLICT OF INTEREST

None declared.

## ETHICAL STATEMENT

This study does not include people and animals.
